# Pathological characterization of hepatic and mesenteric neotropical Echinococcosis in Brazilian Amazonian patients using light and scanning electron microscopy

**DOI:** 10.1590/S1678-9946202567069

**Published:** 2025-10-13

**Authors:** Fernanda Barbosa de Almeida, Alba Cristina Miranda de Barros Alencar, Christiane Leal Corrêa, Eduardo José Lopes Torres, Fernanda Bittencourt de Oliveira, Rosângela Rodrigues-Silva, Nilton Ghiotti Siqueira, Tuan Pedro Dias Correia, José Roberto Machado-Silva

**Affiliations:** 1 Fundação Oswaldo Cruz, Instituto Oswaldo Cruz, Laboratório de Parasitologia Integrativa e Paleoparasitologia, Rio de Janeiro, Rio de Janeiro, Brazil; 2 Universidade Federal Fluminense, Faculdade de Medicina, Departamento de Patologia Clínica, Niterói, Rio de Janeiro, Brazil; 3 Universidade do Estado do Rio de Janeiro, Faculdade de Ciências Médicas, Departamento de Patologia e Laboratórios, Rio de Janeiro, Rio de Janeiro, Brazil; 4 Universidade do Estado do Rio de Janeiro, Faculdade de Ciências Médicas, Departamento de Microbiologia, Imunologia e Parasitologia, Rio de Janeiro, Rio de Janeiro, Brazil; 5 Fundação Hospital Estadual do Acre, Rio Branco, Acre, Brazil; 6 Universidade Federal do Acre, Rio Branco, Acre, Brazil

**Keywords:** Neotropical echinococcosis, Histopathology, Liver, Mesentery, Light microscopy, Scanning electron microscopy, Brazil

## Abstract

Neotropical Echinococcosis (NE) is an emerging parasitic zoonosis of significant public health concern. The disease is caused by the larval stage (metacestodes) of *Echinococcus vogeli* and affects humans in tropical forests of Central and South America. While clinical presentation, radiological imaging, and surgical procedures have been investigated, the pathological features of NE remain partially understood. We performed a comparative study of hepatic and mesenteric metacestodes obtained from Brazilian patients in Acre and Amazonas States during surgical procedures, using both light and scanning electron microscopy for detailed analysis. Liver metacestodes showed three characteristic layers: adventitious, laminated, and germinal. In contrast, mesenteric cysts lacked a consistent layer organization, as the adventitious layer was absent, and the laminated layer was the most prominent membrane within the cyst. Compression exerted by the metacestodes led to hepatic and mesenteric hypertension, characterized by passive hyperemia. Other features induced by hypertension included an expanded sinusoidal bed and extensive areas of hemorrhage resulting from vascular rupture and subsequent blood leakage. In conclusion, the development of mesenteric and hepatic cysts follows distinct patterns, and scanning electron microscopy proves to be a valuable investigative tool for evaluating the pathology of Neotropical Echinococcosis.

## INTRODUCTION

Echinococcosis is a parasitic disease caused by the larval phase (metacestode) of tapeworms from the genus *Echinococcus* and is recognized as a Neglected Tropical Disease (NTD) by the Centers for Disease Control and Prevention (CDC) and the World Health Organization (WHO)^1^. Neotropical echinococcosis (NE) refers to the infection caused by the larval phase of *Echinococcus vogeli* (Cestoda: Taeniidae), which is distributed across tropical forests in Central and South America^2-4^. NE is endemic on the Northern Region of Brazil, which is predominantly covered by the Amazon biome.

*Echinococcus vogeli* (Eg) has a two-host life cycle involving definitive and intermediate hosts, such as wild and domestic canids and rodents, respectively. The natural cycle of Eg occurs when *Speothos venaticus* (bush dog) preys on infected rodents, such as *Cuniculus paca* (lowland paca) and *Dasyprocta agouti* (agouti), which harbor the tissue-invading metacestode (larval) stage. These metacestodes develop into adult tapeworms in the bush dog’s small intestine^4,5^. The domestic cycle involves *Canis familiaris* (domestic dogs), which gain access to infected viscera from hunted wild rodents containing the metacestode^6^. Rodents become infected by ingesting infective eggs in the environment^2,3^, which are a source of infection for humans, who become accidental hosts in NE^2^.

The emergence of NE from wildlife to domestic landscapes is influenced by both economic and cultural factors. In tropical forests, wild game meat serves as a primary source of protein for certain human populations^6-8^. Companion dogs, which are often used in hunting activities, are rewarded with raw viscera from infected pacas, discarded by hunters or their owners^2,7,9^. This practice contributes to establish a domestic NE life cycle, in which pets, similar to wild canids, shed eggs in their feces. Humans are likely to become accidental hosts of NE through the fecal-oral route, considering that they frequently have close contact with contaminated dogs, which are often not dewormed^2,10^.

From an epidemiological perspective, NE is an emerging, neglected, and chronic zoonotic disease of public health concern, particularly affecting populations in areas with limited public health resources and infrastructure^2,11^. Although NE remains underdiagnosed, underreported, underprioritized, and underfunded as a health issue, several reports have indicated a rising number of human cases over time^12-14^.

Like other types of echinococcosis, the juvenile metacestode primarily grows in the liver and lungs, with occasional involvement of other organs in the human body^2,13,15^. The natural history of NE disease comprises multiple developmental stages^16^, with continuous metacestode multiplication, in which the growth rate depends on the host’s localized immune response. Although the incubation period of NE infection varies, it often remains asymptomatic until the metacestodes become large enough to compress host tissues, leading to a range of clinical manifestations, signs, symptoms, and organ damage^17^. In hepatic infections, the right lobe of the liver is most frequently affected. The disease progression is generally slow, with no overt clinical symptoms, but complications can arise in 21% of patients, often due to septic, toxic, or mechanical issues^18^.

The disease is classified into four main forms: Form I (nodular/tumoral), single or few well-defined hepatic lesions, usually at an early stage with favorable surgical prognosis; Form II (multivesicular/pseudotumoral), large masses composed of multiple internal vesicles that compress adjacent structures; Form III (infiltrative), extensive, poorly defined lesions infiltrating hepatic parenchyma and adjacent tissues; and Form IV (disseminated), which is the most advanced stage, with multiple hepatic and extrahepatic lesions (e.g., peritoneum and lungs), associated with higher morbidity and therapeutic complexity^13^.

Most of the current knowledge pertains to the chronic stage of the disease. Typically, patients seek medical attention only in the fourth or fifth decade of life, when the disease has already progressed significantly^2,7,15,19^. As a result, surgical intervention has been demonstrated to be a more effective treatment option compared to other therapeutic approaches^7^.

Despite significant advances recently regarding the chronic form of the disease, particularly in clinical presentation, computed tomography (CT), and magnetic resonance imaging (MRI), the tissue pathology remains partially understood. This study aims to compare histopathological changes in human hepatic and mesenteric tissues, collected from patients undergoing surgery, via analysis using light and scanning electron microscopy (SEM).

## MATERIALS AND METHODS

### Ethics

This project was approved by the Research Ethics Committee - IPEC / Fiocruz, under protocol Nº 0002.1.000.009-04.

This study was conducted at a university hospital (Fundacao Hospital Estadual do Acre [FUNDHACRE], Acre State, Brazil - –9.966, –67.855). Hepatic and mesenteric tissues were collected from eight patients from Acre (–9.974, –67.808) and Amazonas (–3.107, –60.026) during surgery, which was the recommended therapeutic approach due to the severity of the lesions.

Tissue fragments were fixed in 10% formalin at room temperature and subjected to routine histological processing. The blocks were sectioned using a microtome at a thickness of 5 µm. The sections were stained with hematoxylin and eosin (H&E)^20^, Lennert’s Giemsa^21^, Picrosirius^22^, and Masson (Aminoguanidine)^20^. Histopathological analysis and photomicrography were performed using a light microscope (Eclipse E200 microscope and camera – Nikon).

Samples were fixed in 4% buffered formaldehyde and submitted to routine histological processing for the scanning electron microscopy analysis. The paraffin blocks were sectioned into 5 µm slices and mounted on glass slides. The sections were then deparaffinized with xylene in two cycles of 10 min each, fixed onto metallic stubs using carbon tape, and coated with gold (20–25 nm deposition). They were examined using a JEOL JSM-6390 LV scanning electron microscope, operating at 25-30 kV, at the Electron Platform Facility Rudolf Barth (Fiocruz-RJ).

## RESULTS

**Figure 1:**
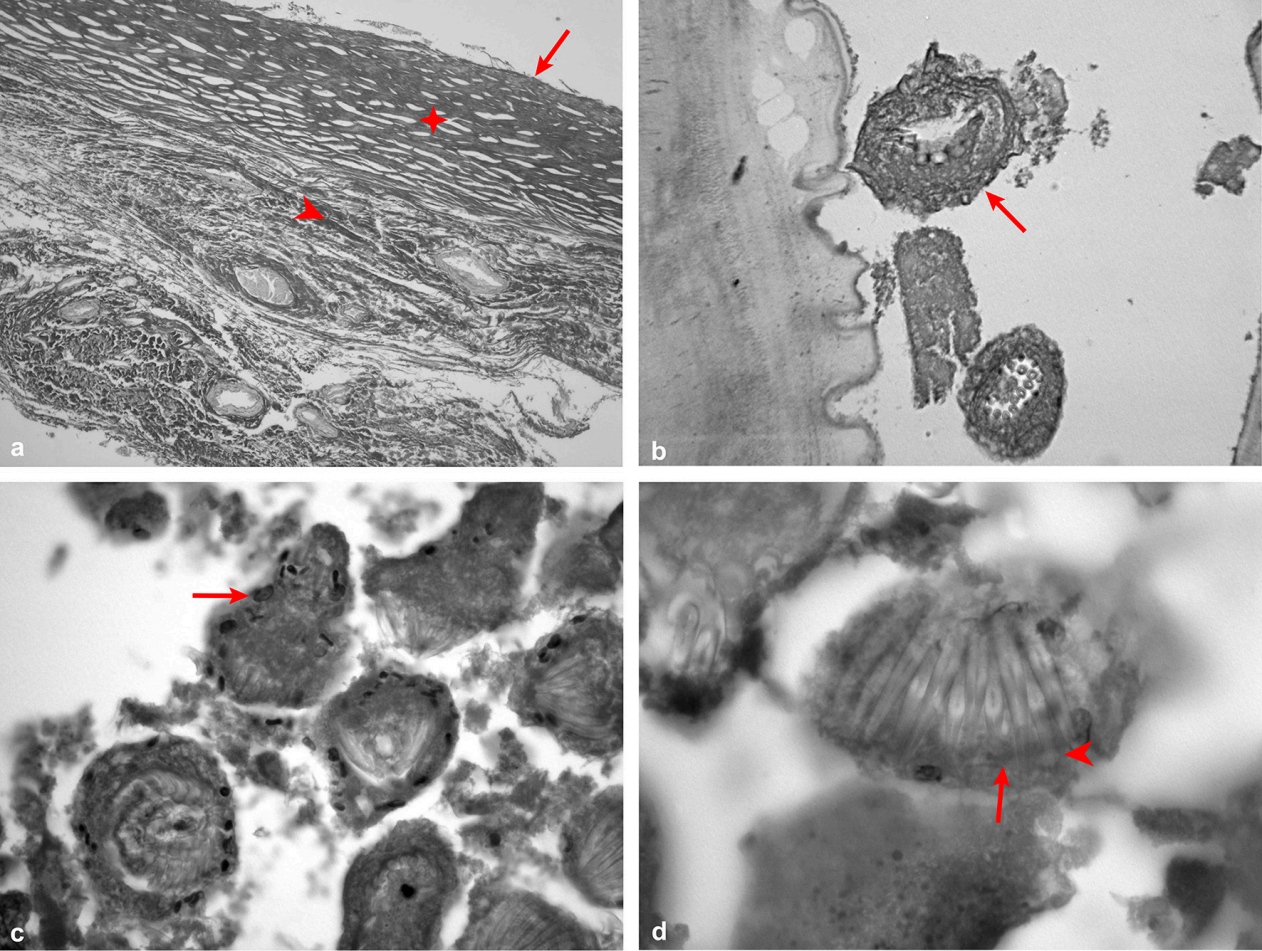
Histopathology of hepatic echinococcosis by light microscopy: (A) Trilaminar structure of *Echinococcus vogeli* polycysts: germinal layer (arrow), laminated layer (✥) and adventitial layer (arrowhead), Picrosirius stain (100x); (B) Protoscoleces in early development (arrow), Picrosirius stain (400x); (C) Protoscoleces full of calcareous corpuscles (arrow), H&E stain (400x); (D) The 1:1 arrangement of hooks in the rostellar pad: large hook (arrowhead) interspersed by a small one (arrow), H&E stain (1000x).

**Figure 2:**
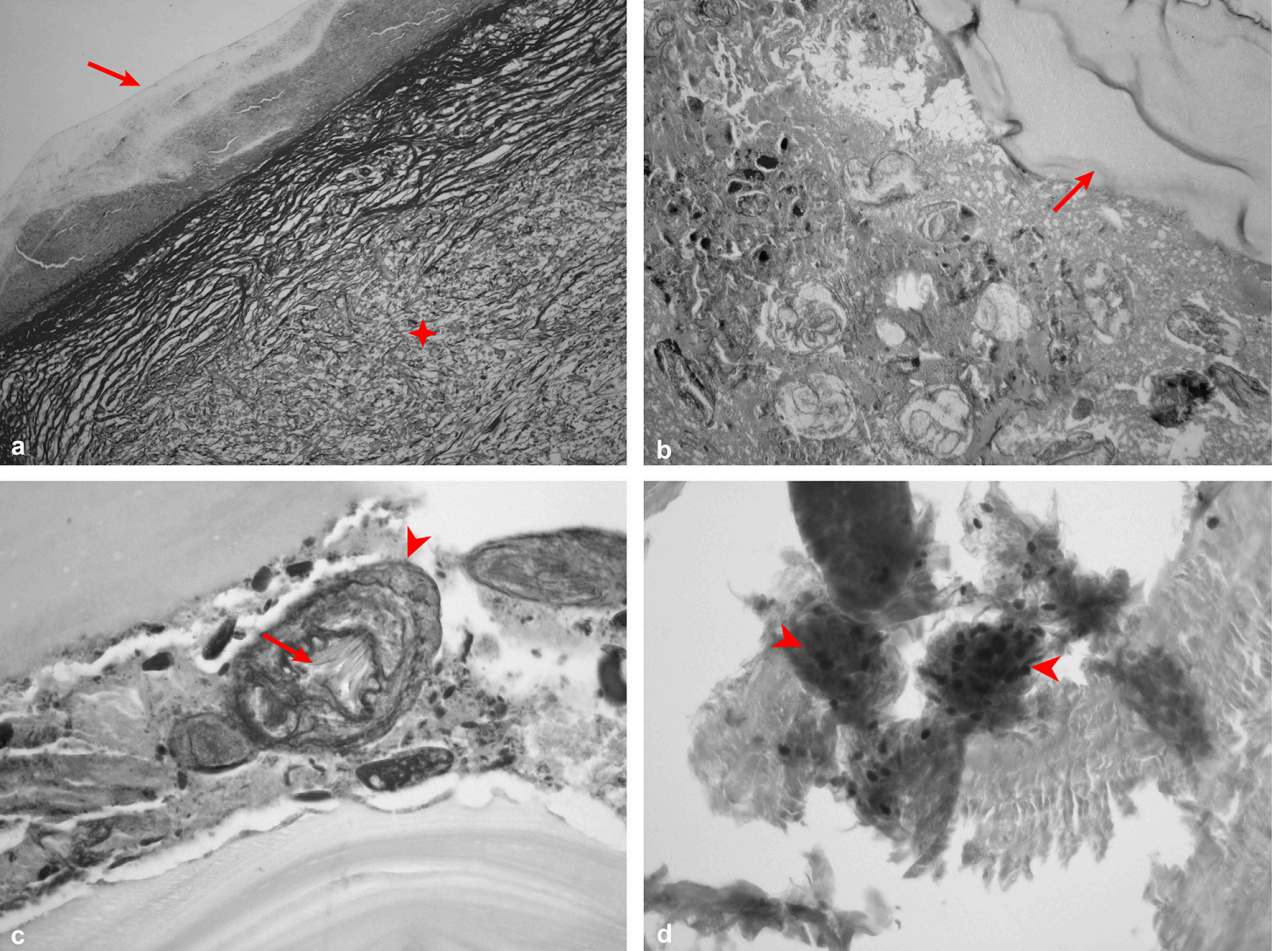
Histopathology of mesenteric echinococcosis by light microscopy: (A) Absence of trilaminar structure of *Echinococcus vogeli* polycysts: germinal layer (arrow) and laminated layer (✥), Picrosirius stain (100x); (B) Proliferative thick germinal layer (arrow), Masson’s trichrome stains (100x); (C) Protoscoleces (arrowhead) with rostellar pad (arrow), Masson’s trichrome stain (400x); (D) Protoscoleces full of calcareous corpuscles (arrowheads), Masson’s trichrome stain (400x).

Light microscopy analysis revealed that all patients had well-structured sterile metacestodes. Hematoxylin and eosin-stained sections showed that liver metacestodes had three characteristic layers: a host-derived adventitial layer, an outer laminated layer (LL), and an inner germinative layer (GL) (Figure 1A). In contrast, mesenteric cysts lacked an organized layering, as the adventitial layer was absent, in which the laminated layer was the most prominent structure in the cyst (Figure 2A). Aside from the absence of the adventitial layer, the remaining cyst structures were present in both hepatic and mesenteric metacestodes, with no discernible differences between them (Figures 1B-D and 2B-D).

The thick germinative layer gave rise to vacuoles (Figure 2B), which eventually form brood capsules and sprout invaginated or evaginated protoscoleces (Figures 1B and 2C), filled with calcareous corpuscles of varying densities (Figures 1C and 2D). The quantity of these corpuscles correlates with the development stage, suggesting that these calcium deposits facilitated the formation of hooks and evagination of the parasite (Figure 2D).

Filled with protoscoleces, the brood capsules protrude from the germinative layer and can rupture, releasing the protoscoleces into the cyst along with the hydatid fluid. This combination of protoscoleces, debris from the cyst layers, and loose hooks is known as hydatid sand. The fluid’s composition is a mixture of molecules derived from both the host and the parasite. The hydatid fluid showed a strong and homogeneous pink coloration in hematoxylin and eosin staining, indicating the presence of proteins within the cysts.

Large quantities of protoscoleces were observed in various development stages. There were both invaginated and evaginated protoscoleces, some had a double row of hooks while others had no hooks at all (Figures 1D and 2C). The protoscoleces with the double row of hooks showed a 1:1 arrangement, featuring a large hook alongside a small hook, and two pairs of dorsal and ventral suckers were also identified.

The host components within the metacestode comprised a fibrovascular tissue featuring a thick outer shell infiltrated by mononuclear cells and eosinophils, along with a few inflammatory layers composed of epithelioid macrophages and giant cells, which are often accompanied by additional mononuclear cells and occasionally eosinophils.

**Figure 3:**
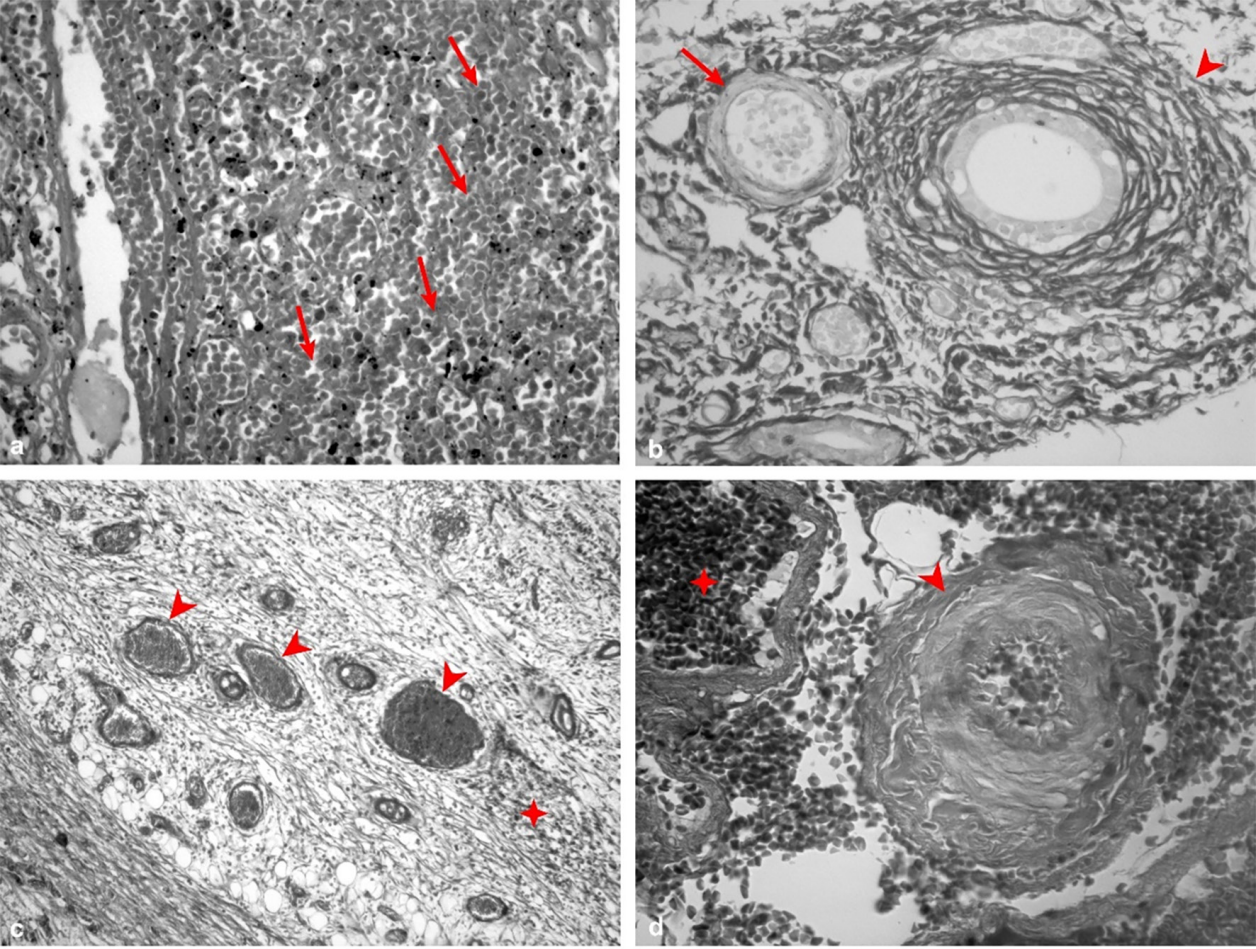
Histopathological changes in hepatic (A, B) and mesenteric (C, D) tissues by light microscopy: (A) Areas of hemorrhage (arrows), H&E stain (400x); (B) Arterioloesclerosis (arrow) and thickening of the venous fibrous layer with collagen (arrowhead), Picrosirius stain (400x); (C) Hemorrhage (✥), passive hyperemia (arrowhead) and formation of new capillaries scattered in the laminated layer (arrow), Picrosirius stain (100x); (D) Arterioloesclerosis (arrowhead) and hemorrhage (✥), Masson’s trichrome stain (400x).

The compression exerted by the metacestodes led to intrahepatic and mesenteric hypertension, characterized by passive congestion due to restricted blood flow in the affected areas. Other alterations included an increase in the density of the sinusoidal beds, which are extensive areas of hemorrhage resulting from blood extravasation due to vascular rupture caused by elevated pressure, and neo-angiogenesis, albeit with sparse and insufficient blood flow in the region (Figure 3).

Areas of the hepatic parenchyma showed hepatocellular necrosis characterized by karyopyknosis, karyorrhexis, and karyolysis, alongside steatosis (both macro- and micro- vesicular). Regions of regeneration featuring binucleated hepatocytes were also observed. Furthermore, the hepatocytes demonstrated hyperplasia and hypertrophy (Figure 3A-B).

Histological analysis of sterile metacestodes revealed a pronounced granulomatous reaction. An inflammatory response adjacent to the laminated layer was noticed, predominantly consisting of epithelioid macrophages, mononuclear cells, and a few eosinophils. Additionally, the sterile metacestodes displayed a degenerated laminar layer resembling hyaline germinal epithelium, which contained brood capsules filled with protoscoleces. The collapse of the germinative layer of the parasite resulted in a tangle of folds within the cyst cavity. The formation of a connective tissue capsule derived from the host was surrounding this structure.

**Figure 4:**
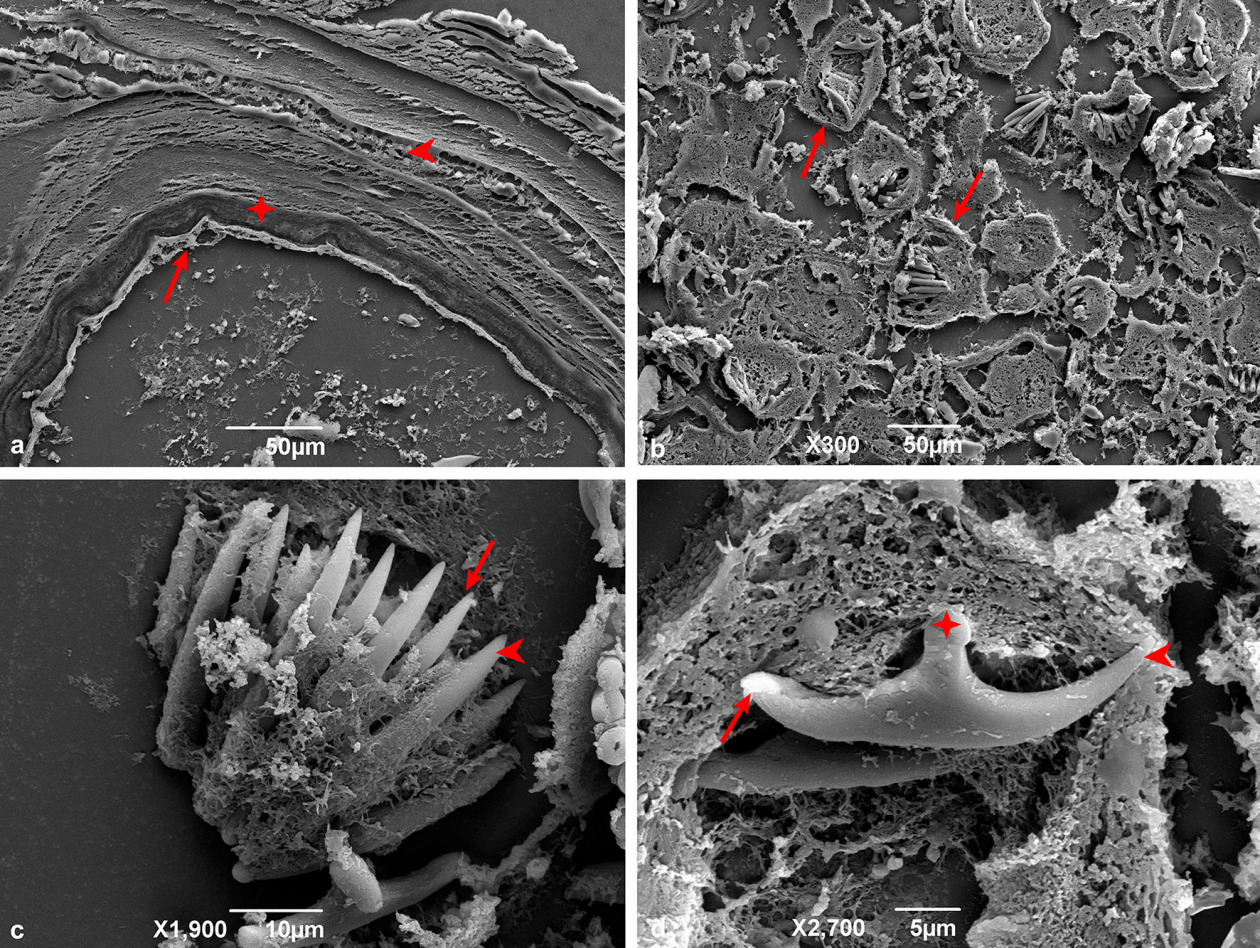
Scanning electron microscopy of histopathology of hepatic echinococcosis: (A) Trilaminar structure of *Echinococcus vogeli* polycysts: germinal layer (arrow), laminated layer (✥) and adventitial layer (arrowhead); (B) Protoscoleces in different development stages (arrows); (C) The 1:1 arrangement of hooks in the rostellar pad: large hook (arrowhead) interspersed by a small one (arrow); (D) Small hook with handle (arrowhead), blade (✥) and guard (arrow).

**Figure 5:**
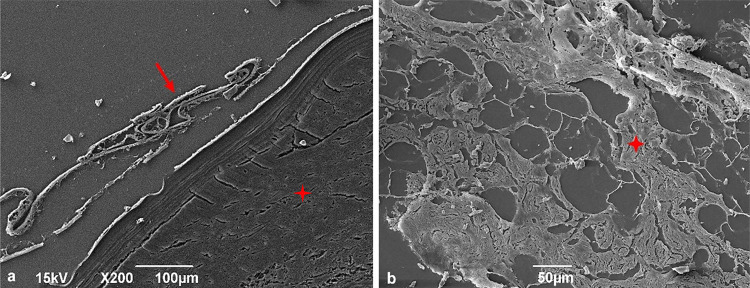
Scanning electron microscopy of histopathology of mesenteric echinococcosis: (A) Absence of trilaminar structure of *Echinococcus vogeli* polycysts: germinal layer (arrow) and laminated layer (✥); (B) Proliferative thick germinal layer (✥).

The scanning electron microscopy analysis corroborated the morphological and histopathological findings obtained via light microscopy. Hepatic metacestodes showed the characteristic three layers (adventitious, laminated, and germinal) (Figure 4A), while mesenteric larvae were devoid of the adventitious layer (Figure 5A). Nonetheless, all other cyst structures were present in both hepatic and mesenteric metacestodes (Figures 4B-D and 5B).

Mesenteric and hepatic cysts showed a thick germinative layer that gave rise to vacuoles (Figure 5B), which subsequently formed the brood capsules, from which protoscoleces emerged. Brood capsules, protoscoleces at various developmental stages (Figure 4B), and free hooklets were observed in the hydatid sand. Scanning electron microscopy (SEM) analysis revealed protoscoleces featuring two rows of hooks on the rostellar pad, with a large hook interspersed between smaller hooks (Figure 4C-D). Furthermore, SEM provided a detailed view of a small hook, showing its handle, blade, and guard (Figure 4D).

**Figure 6:**
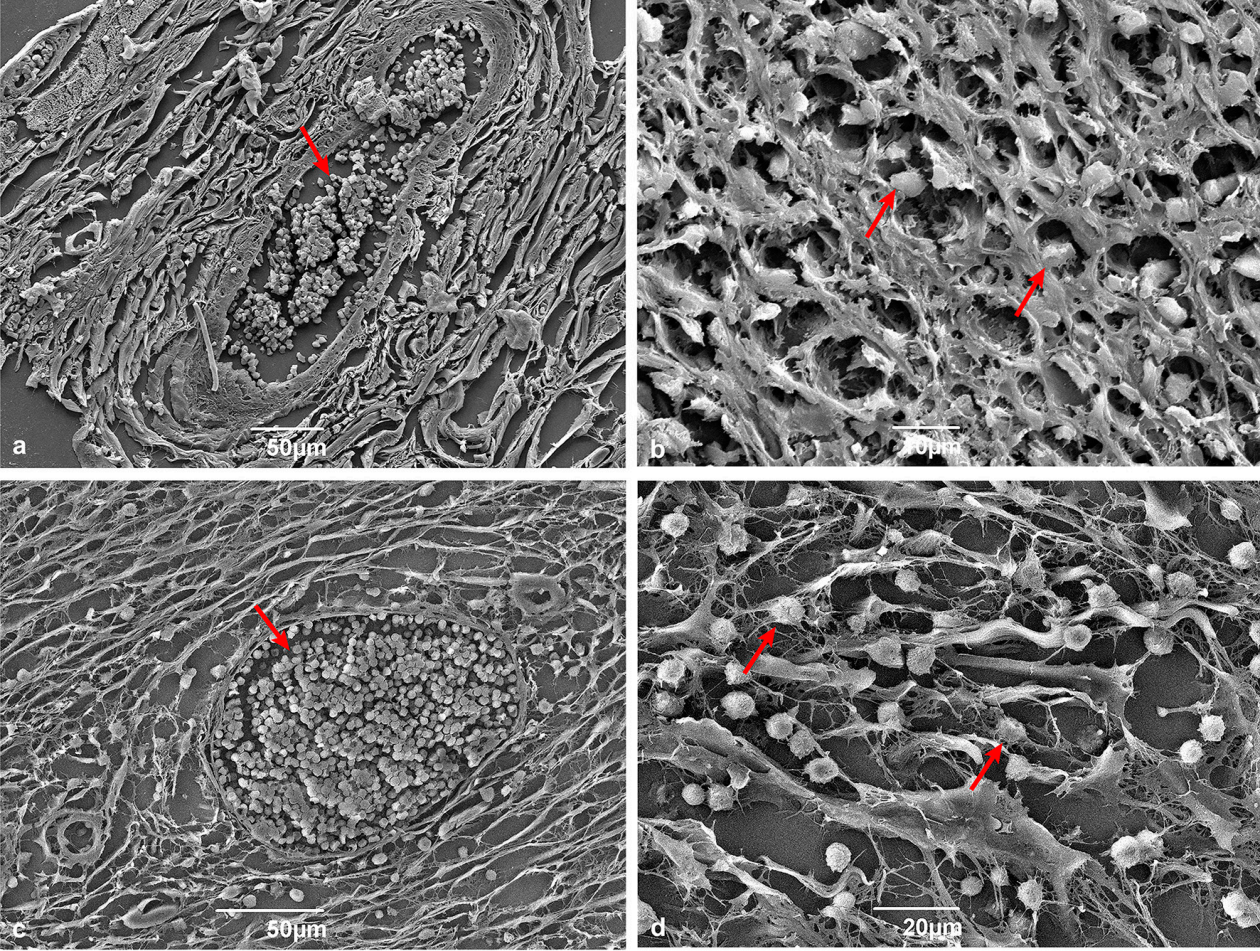
Scanning electron microscopy of histopathological changes in hepatic (A, B) and mesenteric (C, D) tissues: (A) Passive hyperemia (arrow); (B) Areas of hemorrhage (arrows); (C) Passive hyperemia (arrow); (D) Hemorrhage (arrows).

Concerning the histological analysis, the images obtained from SEM corroborated the presence of passive congestion and hemorrhage in both hepatic and mesenteric tissues (Figure 6A-D).

## DISCUSSION

Neotropical echinococcosis is an emerging, neglected zoonotic disease that has seen a rising number of cases. In this study, we reported eight new cases of this zoonosis, all characterized by patients seeking medical care in the advanced stages of infection^2,15,19,23^.

In contrast to cystic echinococcosis, there is limited information on the pathology of neotropical variants. Generally, *Echinococcus* spp. metacestodes cause significant morbidity, in which the chronic form is the most frequently documented^24^.

The most common clinical manifestation in humans is the development of hepatic cysts, which can form in large numbers and grow to substantial sizes, often accompanied by peripheral calcifications. Studies indicate that when the parasite is located centrally, the infection typically shows milder symptoms, but lesions near the hepatic veins or inferior vena cava may lead to a Budd-Chiari-like syndrome. Infections at the liver periphery tend to be less severe, often remaining asymptomatic for extended periods until lesions reach a significant size^25^. Death typically results from complications following major liver resection, septic shock, liver failure, or biliary cirrhosis leading to gastrointestinal bleeding^18^.

In general, patients remain asymptomatic as the disease only produces signs and symptoms when it compresses surrounding organs, making early diagnosis challenging^3^. This, along with its geographic distribution confined to rural areas of rainforests, explains why NE is considered an underdiagnosed zoonotic disease^2,14^. The reported number of human cases (220 up to 2015) likely represents only the tip of the iceberg, as many countries lack comprehensive reporting, and significant challenges persist in diagnosing and treating the disease, particularly in remote areas^14^. Moreover, since NE primarily affects isolated communities, diagnosis often occurs in advanced stages, posing a serious threat to life and contributing to a case fatality rate of approximately 29%^26^.

Literature reports cases of NE in patients from various municipalities in Acre State, Brazil: Sena Madureira^13,23^, Feijo^13^, Assis Brazil^13^, Placido de Castro and Tarauaca^13^, and Pauini, in the Amazon State^13^. Moreover, the clinical form I (involving the liver and mesentery) was the most prevalent^2^, followed by form IV, which exclusively affects the mesentery^13^.

Some studies indicate that the host-origin layer (adventitia) of hydatid cysts has variable thickness depending on the organ in which it is located^27^. Additionally, cyst differentiation has been described in the literature^9^. However, such variations were not confirmed in this study, as the microscopic characteristics of the adventitial layer were consistent across all analyzed cases. Notably, what was observed and should be emphasized is the absence of the adventitial layer in mesenteric cysts.

An outer laminated layer and an inner germinative layer have also been seen by several researchers^28^. Gottstein *et al*.^29^ and Díaz *et al*.^30^ showed that the inner germinal layer represents the living and metabolically active parasite tissue, while the outer, acellular compartment, known as the laminated layer, mediates direct physical contact with the host’s inflammatory response cells.

The composition of hepatic polycysts varies depending on their development stage and the site of infection^9^. Contrary to other reports, it was showed that polycysts could be classified into acute, sub-acute, and chronic phases, with the characteristics of the germinal layer, brood capsules, and proliferative protoscoleces of hepatic polycysts corresponding to these stages^9^.

Previous analyses have revealed a significant presence of plasma cells, lymphocytes, macrophages, and giant multinucleated cells in the adventitial layer and liver parenchyma^31^. We also observed areas of necrosis near intact brood capsules^9^, along with binucleated hepatocytes, indicating hepatic regeneration, as documented in other parasitic helminthic infections. Díaz *et al*.^30^ showed that established infections in humans are frequently surrounded by necrotic areas, with local infiltration of lymphocytes, macrophages, and occasionally eosinophils. This histopathological profile may be linked to the immune and anaphylactic responses elicited by hydatid cysts^31^.

Among the histopathological findings, we highlighted intrahepatic and mesenteric hypertension, characterized by passive hyperemia, hemorrhage, neoangiogenesis, and arteriolosclerosis. Additional features associated with hypertension included enlarged sinusoidal beds and extensive areas of hemorrhage caused by blood leakage due to vascular rupture. Although this observation has not been previously reported, the histopathological finding of hypertension aligns with the primary clinical manifestations described in patients with NE^32^. Hypertension induced by *Echinococcus sp.* has been documented in several reports affecting various organs^33,34^.

The routine microscopy techniques used in pathology include light microscopy and, transmission electron microscopy (TEM), which is less common. However, advancements in scanning electron microscopy (SEM) techniques have enhanced the visualization of biological structures in tissues processed for TEM, particularly via high-resolution scanning electron microscopy (HRSEM)^35^. SEM has been used to analyze metacestode structures^36^, and other studies have demonstrated its effectiveness as a complementary tool for histopathological analysis^37^. The use of SEM to explore ultrastructural details in tissue sections obtained from routine histopathology in parasitology can be especially valuable when biological samples are scarce^38^.

Our findings are consistent with those of Ingold *et al*.^39^, who studied the characterization of the laminated layer of *in vitro* cultivated *Echinococcus vogeli* metacestodes using SEM. The laminated and germinal layers were easily distinguishable, with metacestodes containing brood capsules emerging from the germinal layer, featuring various invaginated or evaginated protoscoleces. Additionally, the morphology of the protoscoleces and rostellar hooks observed in this study aligns with previous descriptions in the literature^36,39^.

SEM is not essential for NE diagnosis but provides valuable detailed information that enhances our understanding of hydatid disease. However, there are some limitations. These methodological challenges—which are common in both basic and clinical studies—are associated with the difficulties of biopsy-based diagnosis and ultrastructural analysis. Although SEM has not yet been widely applied to the pathological study of echinococcosis cases, the promising ongoing development of this technology is expected to aid in solving problems in both research and specific areas of pathology and medical diagnosis^35^.

## CONCLUSION

In conclusion, the development of mesenteric and hepatic cysts follows distinct patterns, and scanning electron microscopy (SEM) has the potential to be a powerful investigative tool in the evaluation of the Neotropical echinococcosis (NE) pathology.

## Data Availability

The complete anonymized dataset supporting the findings of this study is included within the article itself.
